# A Study of Nitrogen Deficiency Inversion in Rice Leaves Based on the Hyperspectral Reflectance Differential

**DOI:** 10.3389/fpls.2020.573272

**Published:** 2020-12-02

**Authors:** Fenghua Yu, Shuai Feng, Wen Du, Dingkang Wang, Zhonghui Guo, Simin Xing, Zhongyu Jin, Yingli Cao, Tongyu Xu

**Affiliations:** ^1^College of Information and Electrical Engineering, Shenyang Agricultural University, Shenyang, China; ^2^Liaoning Engineering Research Center for Information Technology in Agriculture, Shenyang, China

**Keywords:** rice, hyperspectral reflectance difference, nitrogen deficiency, data downscaling, ELM

## Abstract

To achieve rapid, accurate, and non-destructive diagnoses of nitrogen deficiency in cold land japonica rice, hyperspectral data were collected from field experiments to investigate the relationship between the nitrogen (N) content and the difference in the spectral reflectance relationship and to establish the hyperspectral reflectance difference inversion model of differences in the N content of rice. In this study, the hyperspectral reflectance difference was used to invert the nitrogen deficiency of rice and provide a method for the implementation of precision fertilization without reducing the yield of chemical fertilizer. For the purpose of constructing the standard N content and standard spectral reflectance the principle of minimum fertilizer application at maximum yield was used as a reference standard, and the acquired rice leaf nitrogen content and leaf spectral reflectance were differenced from the standard N content and standard spectral reflectance to obtain N content. The difference and spectral reflectance differential were then subjected to discrete wavelet multiscale decomposition, successive projections algorithm, principal component analysis, and iteratively retaining informative variables (IRIVs); the results were treated as partial least squares (PLSR), extreme learning machine (ELM), and genetic algorithm-extreme learning machine (GA-ELM). The results of hyperspectral dimensionality reduction were used as input to establish the inverse model of N content differential in japonica rice. The results showed that the GA-ELM inversion model established by discrete wavelet multi-scale decomposition obtained the optimal results in data set modeling and training. Both the R^2^ of the training data set and the validation data set were above 0.68, and the root mean square errors (RMSEs) were <0.6 mg/g and were more predictive, stable, and generalizable than the PLSR and ELM predictive models.

## Introduction

Rice is one of the world’s three major food crops, ranking second in terms of cultivated area and production. Nearly half of the world’s population depends on rice for food. Globally, Asia, Africa, and Latin America account for 98% of the world’s rice cultivation (Dong et al., 2020). Asia is responsible for 90% of the world’s rice production, and China is the world’s largest rice producer (Wan et al., 2020). As one of China’s major food crops, rice has a wide distribution and large cultivation area (Luo et al., 2020). The use of modern scientific and technological means to achieve high, stable rice yields to ensure food security has become a hotspot of scholarly research (Kawamura et al., 2018; Nutini et al., 2018).

Nitrogen is an important nutrient that affects rice yield (Jiang et al., 2020). The proper application and testing of nitrogen fertilizer to improve fertilizer utilization can improve rice yield and reduce the negative effects of fertilizer on the environment (Zhou et al., 2020). With the development of science and technology, an increasing amount of nitrogen nutrition testing methods have been developed. It is of strategic importance for agricultural development to choose an efficient, non-destructive, and accurate nitrogen nutrition testing method to guide the science-based and reasonable application of fertilizer in rice production systems (Asai et al., 2009; Peng et al., 2009).

Cold land japonica rice is characterized by a slow nutrient release due to low temperatures in the early spring and low ambient and soil temperatures after rice planting. Among the various nutrients, nitrogen has the greatest impact on the growth, development, and yield of rice; it also plays a multifaceted role in maintaining and regulating the physiological functions of rice (Alam et al., 2020; Ding et al., 2020; Sun et al., 2020). Nitrogen deficiency in rice hinders the synthesis of chlorophyll and proteins, thus reducing photosynthesis and affecting dry matter production (Wang et al., 2020). When rice has too much nitrogen, ineffective cuttings increase and the population is prone to overgrowth, resulting in poor light transmission, reduced fruiting rate, delayed maturation, and the increased occurrence of pests, disease, and late fall (Ali et al., 2017).

In recent years, with the development and application of hyperspectral technology, there have been great strides in agricultural information technology for monitoring crop growth and estimating yields, significantly improving the scientific nature of crop production dynamic detection and management decisions (Zhang et al., 2011, 2015, 2016). During crop development, changes in nitrogen nutrient levels cause changes in leaf color, chlorophyll level, and moisture content that lead to hyperspectral changes, which is the theoretical basis for nitrogen estimation using hyperspectral technology. Yu et al. (2016) combined the radiative transfer model and Gaussian process regression model to determine the crop leaf chlorophyll content and effectively monitored the nitrogen nutrition of rice. Due to the high data dimension of the hyperspectral information, it is usually necessary to perform a downscaling of the hyperspectral data before using the downscaled results to build a quantitative inversion model with the nitrogen content (Chu et al., 2014). In order to accurately estimate the vertical distribution of nitrogen in the leaves of rice plants, He et al. (2020) constructed a vertical distribution model of leaf nitrogen content using hyperspectral data by the vegetation index method combined with the height of rice plants to provide a technical basis for the vertical distribution of nitrogen in rice.

Due to the special soil background, climate conditions, and growth period in cold regions, it is necessary to find adaptable models to assess rice growth and nutrient availability (Yu et al., 2017; Sun et al., 2020). The support vector regression based on the binary particle swarm optimization algorithm (BPSO-SVR) method was used to estimate the nitrogen content of rice at different growth stages using the cold land japonica rice canopy hyperspectral reflectance, which can be effectively used to monitor the nitrogen status of rice (Tan et al., 2018). Artificial neural networks (ANNs) are learning, fault-tolerant, and real-time, and have unparalleled advantages for fitting non-linear problems. ANNs can provide effective technical and theoretical support for many fields. Current research on ANNs in hyperspectral inversion of crop nitrogen is also increasing (Zhu et al., 2017; Li et al., 2018; Wen et al., 2019; Yang et al., 2019).

Many researchers have studied the inversion of rice nitrogen content using hyperspectral techniques, but the nitrogen content alone cannot directly guide the quantitative and accurate fertilization of rice in the production process (Din et al., 2019; Ge et al., 2019; Li et al., 2019; Zhang et al., 2019; Liu et al., 2020; Wang et al., 2020). Ulrich (1952) was the first to introduce the concept of critical nitrogen concentration, or the minimum nitrogen concentration required for a crop to reach maximum dry matter, and developed a dilution model of critical nitrogen concentration as a power function using plant dry matter and plant nitrogen concentration (Linquist et al., 2013; Zhao et al., 2016). With the development of research on rice growth and nitrogen nutrition, some researchers have shown that the nitrogen content of plants decreases gradually as the plants grow and develop, even at adequate nitrogen levels, and that this trend eventually affects rice yield (Jalloh et al., 2009; Zhang et al., 2009; Ye et al., 2013). In the early reproductive stage, rice plants are independent of each other and there is no competition for light between rice plants. With the increase of plant biomass, the decrease of nitrogen concentration is not obvious, so the nitrogen concentration in the early reproductive stage of rice is relatively stable. However, as the amount of nitrogen applied increases, the leaf area index, biomass, and other indicators related to nitrogen uptake in rice also increase, which results in a situation where the nitrogen content varies with biomass while the nitrogen concentration is the same (Prasanna et al., 2012; Aasen et al., 2014; Arai-Sanoh et al., 2014; Pittelkow et al., 2014; Geng et al., 2015). Some studies have guided rice fertilization by constructing different nitrogen concentration curves, but different regions and varieties all need to construct separate critical nitrogen concentration dilution curves to better guide rice fertilization, and critical nitrogen concentration curves are cumbersome to construct and require strong agricultural knowledge to establish (Matsunami et al., 2009; Zeng et al., 2012; Qin et al., 2013; Zhao et al., 2015; Wang and Peng, 2017).

To address the current scientific need, we used hyperspectral reflectance information to construct a model and theoretical reference for the difference in hyperspectral reflectance of different rice leaves and the corresponding difference in the nitrogen content to establish a rice nitrogen difference inversion model that will support improved implementation of accurate fertilization practices in rice cultivation in cold regions.

## Materials and Methods

### Study Area and Experimental Details

The experiments were conducted in June−September 2018 and 2019 at Liutianhe Village (123°63′E, 42°01′N), Qingshuitai Town, Shenyang New District, Shenyang, Liaoning Province, China. The tested rice variety was “Gengyou 653.” There were four nitrogen (N) fertilizer gradient treatments in the test field: N0, N1, N2, and N3. N2 was the local standard N application rate; the N3 and N1 experimental application rates were increased and decreased by 50% of N2, respectively. The four different N application rates were N0 (without N), N1 (50 kg/ha), N2 (100 kg/ha), and N3 (150 kg/ha). Each treatment was replicated four times for a total of 16 test plots. The mass fractions of total N and quick-acting N in 0–0.5 m tillage soil in the test field were 154 and 104.032 mg/kg, respectively, and the rest of the field is managed under high-yield cultivation. Data were collected at the rejuvenation, tillering, and tasseling stages. A total of 259 valid samples were obtained, of which 189 groups were training sets and 79 groups were validation sets (as shown in Table 1).

**TABLE 1:** Statistical table of N content in rice leaves.

Sample set	Samples no.	Minimum value (mg⋅g^–1^)	Maximum value (mg⋅g^–1^)	Mean value (mg⋅g^–1^)	Standard deviation (mg⋅g^–1^)
Total	259	1.060	4.874	2.897	0.926
Training set	189	1.060	4.874	2.926	0.935
Validation set	70	1.125	4.689	2.822	0.899

### Data Acquisition

#### Measurement of Hyperspectral Reflectance of Rice Leaves

We used a leaf clip (model A122325, manufacturer ASD Inc.). The incidence angle of light was 0° from normal. The main measurement was the middle position of the leaf, and 10 measurements per leaf were averaged as the hyperspectral reflectance of that leaf. The hyperspectral reflectance calibration was performed every 5 min during the measurement. The blade clips were on a black background to ensure reproducibility in the acquisition of hyperspectral reflectance data from rice leaves.

#### Measurement of N Deficiency in Rice Leaves

Rice was destructively sampled in each plot, brought back to the laboratory, and all of the fresh leaves from the point were cut off and placed in an oven at 105°C for 30 min and dried to a constant amount at 65°C. The rice was weighed and crushed, and the ground powder was placed into a labeled self-sealing bag for testing the N content of the blades (N, mg/g) using the traditional Kjeldahl method for N determination.

The inversion model of N deficiency in cold land japonica rice is based on the construction of a database of differential spectral reflectance and differential N content, and the formulation of standard spectral reflectance and standard N content is key to the construction of the database. Since the objective of this study was to provide a reference for accurate fertilizer application without yield loss, the model is based on the principle of applying a critical amount of N fertilizer that is constant with increasing N fertilizer application, using the standard hyperspectral reflectance to ascertain the standard plant N content.

During rice harvest, a square frame made of PVC pipe with a side length of 1 m was randomly placed into the plot to be measured to calculate the total number of holes of rice within 1 m^2^, and then calculate the effective panicle number of rice per hole, and finally calculate the grain number per ear and the mass of 1,000 grains. As shown in Table 2, the rice fields with N2 application had the highest yield with 387.15 kg/667 m^2^. According to the principle of highest yield, the plots with N2 application were defined as standard fields, the average of all of the spectra collected in the plots was defined as standard spectra, and the average N content in the plots was defined as the standard N content. The difference between the N content of rice leaves collected from non-standard plots and the standard N content was calculated to determine the N deficiency in the rice leaves.

**TABLE 2:** Statistical table of rice yield.

Nitrogen level (kg⋅hm^–2^)	Total	Effective number of spikes	Effective number of grains	Yield (kg⋅667 m^–2^)
0	15.8	11.00	90.4	261.99
50	16.2	12.85	102.4	342.66
100	16.0	14.60	104.4	387.15
150	15.9	13.46	102.5	333.60

#### Obtaining the Difference of Hyperspectral Reflectance

**FIGURE 1:**
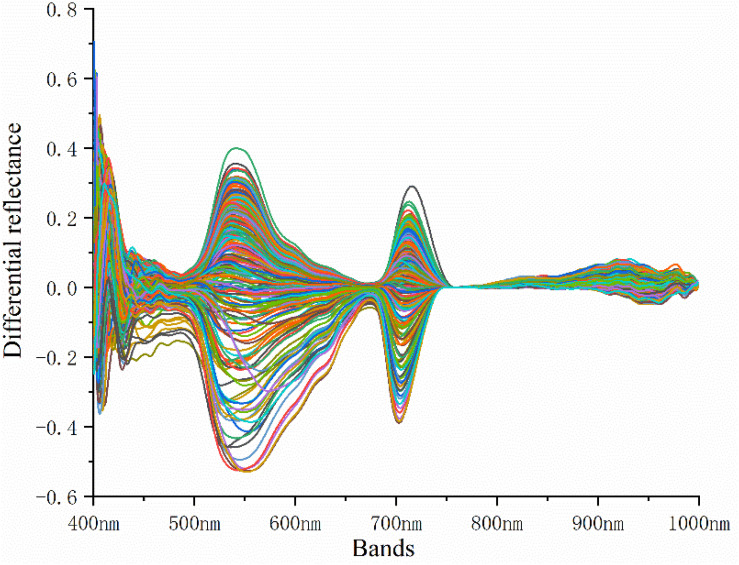
Spectral reflectance difference.

After acquiring the hyperspectral reflectance of rice leaves, the difference between the spectral reflectance and the standard spectral reflectance of rice leaves collected from non-standard plots was used to determine the difference in the spectral reflectance. The spectral reflectance difference is shown in Figure 1.

#### Hyperspectral Differential Data Reduction Method

The full-band hyperspectral differential data contain a lot of redundant information. If the full hyperspectral differential was used as the input in the modeling process, the model error increased and the inversion was not effective. Therefore, feature extraction of hyperspectral difference information was needed to reduce the data dimension and extract useful hyperspectral difference feature data from the high-dimensional information as input data for building inverse models.

In this study, we used the discrete wavelet multi-scale decomposition (DWMD), principal component analysis (PCA), successive projections algorithm (SPA), and iteratively retaining informative variables (IRIV) for spectral reduction. Hyperspectral data reduction mainly includes hyperspectral feature extraction and feature band selection. The PCA is a commonly used method for hyperspectral feature selection. The DWMD discretizes the scales and displacements in the continuous wavelet transform and combines them with the distribution of the wavelet signal energy on each scale to compress the spectral signal dimensions, reduce the number of characteristic bands, and highlight the spectral profile information. The SPA and IRIV are commonly used methods for hyperspectral feature band selection.

#### Discrete Wavelet Multi-Scale Decomposition Method

The DWMD can accurately decompose the spectral signal in the time domain and frequency. The domain for leaf spectral information, or the transformation of the signal in the time domain, is equivalent to the transformation of the spectral data in the spectral band. Thus, the wavelet basis function can be expressed as a degree decomposition:

(1) $$\phi_{a,b}\,\left(\lambda \right)=\frac{1}{\sqrt{a}}\,\phi \,\left(\frac{\lambda -b}{a}\right)\,a,b\in R;a>0;\int_{-\infty }^{+\infty }\phi \,\left(\lambda \right)\,d\lambda =0$$

where *a* is the telescoping factor, *b* is the panning factor, λ is an independent variable, and the function mean is 0.

The discrete wavelet transformation is the discrete form of the decomposition scale and panning, and it is a one-dimensional input signal. The discrete wavelet transform coefficient *W*_*j*,*k*_ is the approximation of the base function to the signal after discrete scaling and panning, and can be expressed by Eq. 2:

(2) $$W_{j,k}=\left(f\,\left(\lambda \right),\phi_{j,k}\,\left(\lambda \right)\right)$$

where the wavelet function ϕ_*j*,*k*_(λ) can be calculated by Eq. 3:

(3) $$\phi_{j,k}\,\left(\lambda \right)=2^{\frac{-j}{2}}\,\phi \,\left(2^{-j}\,\lambda -K\right)$$

where *j* and *k* are the jth decomposition and kth wavelet coefficients, respectively, and the scale of discrete wavelet variation is usually taken as a binary sequence where *j* = 2,4,8…to make the calculation more efficient. Multi-scale decomposition of the signal is based on a discrete wavelet transform algorithm, the decomposed wavelet coefficients are the approximate coefficients for recording low frequency signals, and the detail coefficients are for recording high frequency detail signals. The wavelet approximation coefficient results in the input to the inverse model.

#### Successive Projections Algorithm

A successive projections algorithm is a forward variable selection algorithm that minimizes the co-linearity of vector space and is now widely used in biomedical imaging, computed tomography, signal processing, and spectroscopy. The SPA algorithm is divided into three stages. In the first stage, several subsets of alternative wavelength variables with the smallest covariance are screened out. Assuming that the initial variable position, *k*(0), and the number of variables, or *N*, have been given, the specific steps of this stage are as follows:

Step 1: Before the first iteration (*n* = 1), assign column *j* of the training set spectral matrix *X*_*cal*_ to *j = 1*…, *J.*
Step 2: Make S the set of all unselected wavelength variables, that is, *S* = {j| 1 ≤ j ≤ J and j {k(0)…, k(n−1)}}.
Step 3: For all j ∈ S, compute the projection on the subspace orthogonal to *X*_*k(n–1)*_:

(4) $${\mathrm{PX}}_{j}=X_{j}-\left(X_{j}^{T}X_{k\,\left(n-1\right)}\right)X_{\left(n-1\right)}\left(X_{k}^{T}X_{k\,\left(n-1\right)}\right)X_{k\,\left(n-1\right)}){}^{-1}$$

where *P* is the projection operator.

Step 4: Record the position of the wavelength with the largest paradigm of projected value:

(5) $$k\left(n\right)=\arg\left(\max||{\mathrm{PX}}_{j}||,j\in S\right)$$

Step 5: Make *X*_*j*_ = *PX_j_*,*j*∈*S*.
Step 6: Make *n* = *n* + 1If *n* < *N* then return to step 2.
End: Obtain *N* alternative wavelength positions: {*k*(*n*);*n* = 0,…,*N*−1}.

The number of projection operations performed during the selection process is (*N*−1)(*J*-*N*/2).

In the second stage, multivariable linear regression models were built using variables from each subset separately, and the smallest subset of the root mean square error (RMSE) was selected.

In the third stage, stepwise regression modeling was performed on the subset selected in the second stage to obtain a set with a smaller number of variables, that is, one with minimal loss of predictive accuracy. The wavelength variable in this set is the selected effective wavelength.

#### Principal Component Analysis

Principal component analysis recombines the original variables into a new set of several unrelated composite variables summed to reflect as much information as the original variables for the possible statistical method. PCA is an attempt to replace the original variables with a new set of unrelated composite indicators by regrouping the original set of indicators with a certain degree of correlation (e.g., P indicators). The usual mathematical treatment is to combine the original P indicators in a linear fashion as a new composite indicator. The most classic approach is to express this in terms of the variance of F1 (the first linear combination selected, that is, the first composite indicator); thus, the larger the Va (rF1), the more information that F1 contains. Therefore, the F1 selected in all of the linear combinations should be the one with the highest variance, so F1 is called the first principal component. If the first principal component is not enough to represent the information of the original P indicator, then F2 may be used to choose the second linear combination, in order to effectively reflect the original information. The existing F1 information does not need to appear in F2 again; expressed in mathematical language this is the requirement of *Cov* (F1,F2) = 0, then F2 is called the second principal component, and so on can be used to construct the third, fourth,…, P, or principal component.

#### Iteratively Retaining Informative Variables

The IRIV algorithm uses random combinations of variables and takes full account of the interactions between variables while generating a binary matrix of random combinations of all of the variables (random combinations of behavioral variables, listed as number of variables) on the basis of a binary matrix rearrangement filter. A partial least squares model is then built based on each row of the matrix (i.e., random combinations of variables) separately, and evaluates the model effect of different random variable combinations using a cross-validation root mean square error (RMSECV). Based on the model clustering analysis method, the RMSECV averages for each wavelength variable are calculated with and without this variable, and the difference in mean values between the difference of mean values and the non-parametric Mann-Whitney *U* test *P*-values are obtained to determine the importance of this variable.

#### Inverse Modeling Methods

In this study, we used three methods to test the accuracy and reliability of the model and to select the optimal inverse model of rice leaf N deficiency based on the decision coefficient R^2^ and RMSE of the model: the partial least squares regression (PLSR), extreme learning machine (ELM), and genetic algorithm-extreme learning machine (GA-ELM).

ELM is widely used in many fields due to its fast learning speed and small training error. However, the algorithm randomly generates the connection weights between the input and implicit layers and the thresholds of the implicit layer neurons, and no adjustment is required during the training process, resulting in poor stability and generalization of the inverse model built by the algorithm. In this study, a genetic algorithm based on the principles of evolutionary superiority, natural selection, and survival of the fittest was used to optimize the ELM.

Specific implementation steps for genetic algorithm optimization training are detailed below.

(1) An initial population is generated randomly *X*_*m×l*_, where *m* is the initial population number, individual length *l* represents both the number of gene values for each individual and the initial weight of a neural network, and the gene values in an individual correspond to the initial weight of the neural network one to one. In this study, real number coding was used to encode the gene values, which avoids the decoding process and improves the training efficiency:

(6) $$l=s_{1}\,s_{2}+s_{2}\,s_{3}+s_{2}+s_{3}\;$$

where *l* is the individual length, *s*_*1*_ is the number of input layer nodes, *s*_*2*_ is the number of implicit layer nodes, and *s*_*3*_ is the number of output layer nodes.

(2) The genetic algorithm sorts individuals in the initial population with larger adaptation values into subpopulations to continue optimization training by calculating the output error value *E*_*i*_ and the adaptation value *f*_*i*_ for each individual in the initial population, and evaluating the size of the individual adaptation value *f*_*i*_:

(7) $$f_{i}=\frac{1}{1+E_{i}}$$

**FIGURE 2:**
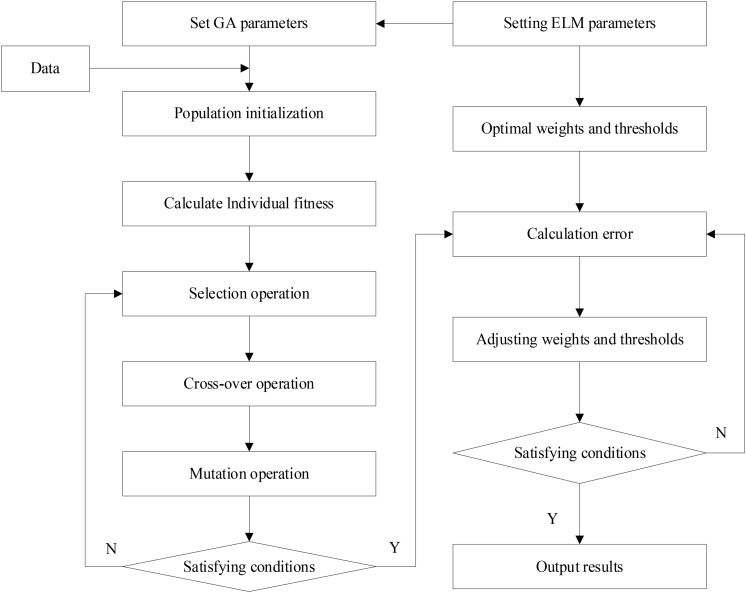
Flow chart optimization of ELM based on GA.

(3) In each subpopulation, the probability that the *i* individual selected will perform a crossover or mutation operation is *p*_*i*_, and whether this individual needs to perform crossover or genetic operation is judged by the adaptive function of crossover rate *p*_*c*_ and mutation rate *p*_*m*_, and the value of *p*_*c*_ and *p*_*m*_ will change adaptively according to the size of the adaptive value *f*_*i*_ of the individual. This avoids the problems of search randomization, slower search speed, loss of important genes of antibodies, and reduced probability of generating new individuals caused by *p*_*c*_ and *p*_*m*_ values that are too high or too low, keeping the population perpetually diverse:

(8) $$p_{i}=f_{i}/\sum_{i=1}^{m}f_{i}$$

(9) $$p_{c}=\left\{ \begin{array}{cc} \frac{k_{c}\,\left(f_{\text{max}}-f_{c}\right)}{\left(f_{\text{max}}-\bar{f}\right)}, & f_{c}\geq \bar{f}\\ k_{c}, & f_{c}<\bar{f} \end{array}\right.$$

(10) $$p_{m}=\left\{ \begin{array}{cc} \frac{k_{m}\,\left(f_{\text{max}}-f_{m}\right)}{\left(f_{\text{max}}-\bar{f}\right)}, & f_{m}\geq \bar{f}\\ k_{m}, & f_{m}<\bar{f} \end{array}\right.$$

where *k*_*c*_ and *k*_*m*_ are all real numbers <1, *f*_*c*_ is the individual fit value to be crossed, *f*_*m*_ is the individual fit value to be mutated, *f*_*max*_ is the upper bound of the fit value *f*_*i*_, and $\bar{f}$ is the mean of the fit value *f*_*i*_. The ELM flowchart based on the genetic algorithm (GA) optimization is shown in Figure 2.

In this study, the RMSE and coefficient of determination (R^2^) were used as evaluation criteria for assessing the accuracy of the hyperspectral remote sensing inversion model.

## Results and Analysis

### Selection of Hyperspectral Features and Characteristic Bands

#### PCA Extracts Hyperspectral Difference Features

In this study, five principal components of hyperspectral reflectance difference were extracted by PCA in the range of 400–1,000 nm. These five principal components were the input variables of the model.

#### Extraction of Hyperspectral Difference Features by DWMD

The determination of the wavelet master function and the optimal decomposition scale is one of the key aspects of the wavelet transform for feature extraction. If the decomposed wavelet information can both reflect the profile characteristics of the spectrum and achieve the purpose of data compression, then the wavelet master function and the decomposition scale can be considered the best choice.

**FIGURE 3:**
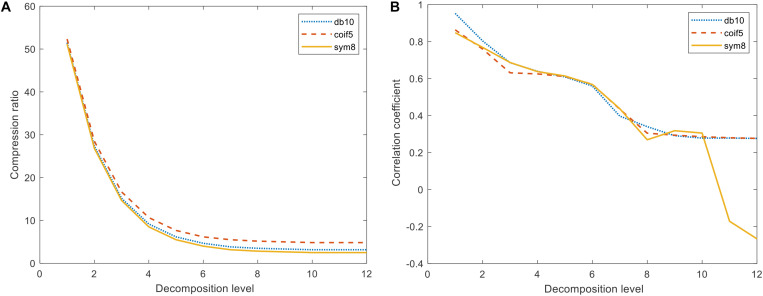
Compression ratio and correlation coefficient under different wavelet generating functions. **(A)** Variation of compression ratio with the number of decomposed layers. **(B)** Variation of correlation with the number of decomposition layers.

For the difference spectra, discrete wavelet transformations were applied to the db10, coif5, and sym8 wavelet functions on two *j* (*j* = 1,2,…,12) scales and were recorded as scales 1 − 12 (levels 1 − 12). After 12 levels of DWMD of the spectra, the number of approximation coefficients obtained from each layer classification was extracted, and the curve of the proportion of the number of decompositions with the number of layers was obtained, as shown in Figure 3A. The wavelet approximation signal characterized the profile of the spectrum, the signal reconstruction of each layer of approximation coefficients under different wavelet parent functions was performed, and the correlation coefficients of each reconstructed spectral signal with the original spectral signal were calculated as shown in Figure 3B and Table 3.

**TABLE 3:** Number of decomposition level under different wavelet generating functions.

	db10			coif5			sym8		
			
Decomposition level	Relevance	Approximate number	Compression ratio	Relevance	Approximate number	Compression ratio	Relevance	Approximate number	Compression ratio
1	0.952	309	51.500	0.864	314	52.333	0.848	307	51.167
2	0.804	164	27.333	0.760	171	28.500	0.769	161	26.833
3	0.686	91	15.167	0.631	100	16.667	0.687	88	14.667
4	0.639	55	9.167	0.625	64	10.667	0.638	51	8.500
5	0.609	37	6.167	0.611	46	7.667	0.614	33	5.500
6	0.560	28	4.667	0.569	37	6.167	0.568	24	4.000
7	0.397	23	3.833	0.435	33	5.500	0.437	19	3.167
8	0.340	21	3.500	0.305	31	5.167	0.269	17	2.833
9	0.291	20	3.333	0.294	30	5.000	0.318	16	2.667
10	0.278	19	3.167	0.286	29	4.833	0.306	15	2.500
11	0.279	19	3.167	0.280	29	4.833	−0.171	15	2.500
12	0.277	19	3.167	0.277	29	4.833	−0.266	15	2.500

As shown in Figure 3, in the decomposition of the three wavelet mother functions in the 7–12 layers, the correlation coefficient change law was consistent with the other two types of wavelet mother functions as a whole. When the number of decomposed layers reached 10, the number of approximate coefficients eventually stabilized. Compared with the other two classes of parent functions, coif5 had the highest number of wavelet approximation coefficients and the weakest data compression, while sym8 had the strongest wavelet parent function data compression. As can be seen in Table 3, the sym8 wavelet parent function had the lowest number of approximation coefficients and the highest correlation coefficients after decomposition at the 10th level. Therefore, considering the data compression and the ability to preserve the original spectrum, we determined that the sym8 wavelet master function works best when decomposed at layer 10.

#### SPA Selection of Valid Feature Bands

**FIGURE 4:**
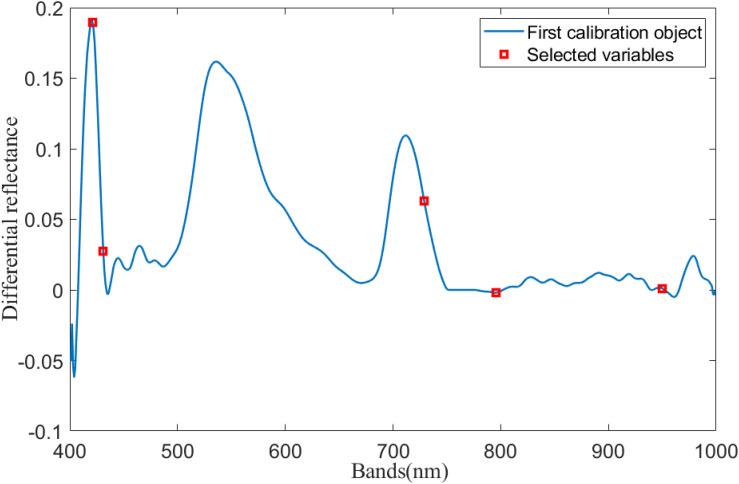
Corresponding spectral band.

A continuous projection algorithm was used for the selection of spectral signature bands for rice leaf hyperspectral difference calculation. As shown in Figure 4, the optimal number of spectral bands was determined to be five based on the internal cross-validation RMSECV values of the correction set.

As can be seen in Figure 3, five characteristic bands were selected from the 400- to 1,000-nm bands using SPA: 421, 431, 729, 796, and 950 nm. The difference in reflectance at the selected feature band was used as an input to the inverse model.

#### IRIV Selection of Valid Feature Bands

**FIGURE 5:**
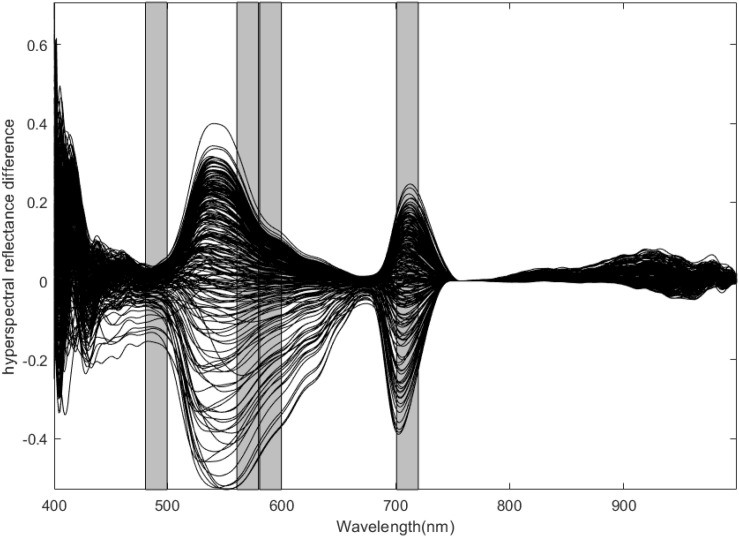
IRIV characteristic band interval.

In this study, 599 spectral variables of the whole band were divided into 30 intervals according to 20 bands using a synergy interval PLS method. Four interval combinations were set, the RMSE minimum value was selected by 10-fold cross validation to determine the optimal joint interval, and the combination of the 5th, 9th, 10th, and 16th intervals was finally determined as the optimal joint interval with corresponding wavelength regions of 481–500 nm, 561–580 nm, 581–600 nm, and 701–720 nm, respectively. Figure 5 shows the position of m in the full band spectrum. Full-band spectra were reduced from 599 to 80 spectral variables by the synergy interval PLS method, and then reduced to 44 variables by five iterations of IRIV and the elimination of 8 variables in reverse, resulting in 36 characteristic variables. The characteristic wavelengths are as follows: 481, 482, 484, 485, 486, 487, 488, 489, 492, 493, 494, 497, 498, 499, 500, 562, 564, 566, 581, 583, 585, 587, 589, 590, 594, 595, 596, 599, 600, 712, 713, 714, 716, 717, 719, and 720 nm.

Several of the above 36 variables belonged to adjacent bands, and band was highly significant in a correlation test (*P* = 0.01; correlation coefficients were all >0.9). One goal of this study was to remove wavelengths that are correlated with low differences in N concentration; we ultimately retained the wavelengths of 500, 566, 600, and 712 nm as those that are strongly correlated with N concentration in the modeling process.

#### GA-ELM Inversion Model for Rice N Deficiency

**FIGURE 6:**
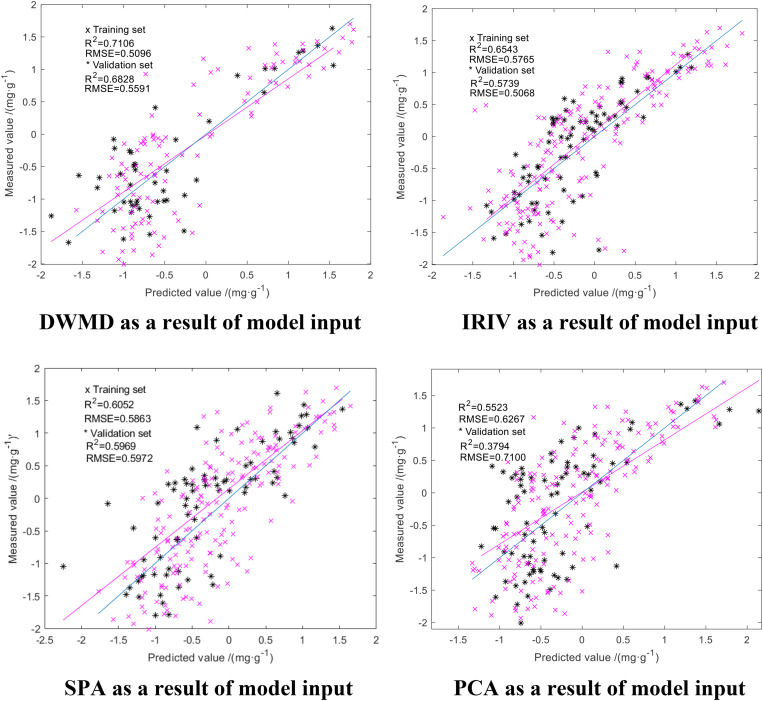
GA-ELM modeling results.

We downscaled the obtained spectral reflectance of the difference using SPA, PCA, DWMD, and IRIV. The results of the four methods were used as the input to the model, with the actual measured N deficiency of rice leaves as the output. The parameters of GA-ELM were determined by repeated tests: the activation function is *Sigmoid*, the output function is *Purelin*, the practice function is *trainlm*, the crossover probability = 0.5, the variance probability = 0.5, and the decision coefficient R^2^ and the RMSE were used as evaluation *criteria* for the model. The modeling results are shown in Figure 6.

As shown in Figure 6, the results obtained from the four dimension-reduction methods were used as an input in GA-ELM inversion modeling, and the inversion modeling results were consistent. The R^2^ of training set and verification set were both above 0.555, and RMSE was below 0.8 mg/g. Among them, the GA-ELM model with the approximation coefficients of wavelet decomposition obtained by discrete wavelet multiscale decomposition had the highest accuracy with an R^2^ for the training set and verification set of 0.7106 and 0.6828, respectively, while the RMSE was 0.5096 and 0.55591 mg/g, respectively. The accuracy of the GA-ELM model established using the result of dimension reduction of IRIV was good. The R^2^ of the training set and validation set was 0.6543 and 0.5739, respectively, and the RMSE was 0.6543 and 0.5066 mg/g, respectively. The GA-ELM model built with PCA dimension reduction results had the lowest accuracy. The R^2^ of the training set and validation set was 0.5523 and 0.3794, respectively, and the RMSE was 0.6267 and 0.7100 mg/g, respectively. The precision of the GA-ELM model built with the results of dimension reduction of SPA was between the two; the R^2^ of the training set and validation set was 0.6052 and 0.5969, respectively, and the RMSE was 0.5863 and 0.5972 mg/g, respectively. Thus, GA-ELM improves the stability and prediction ability of the model.

#### Comparison With Other Inversion Models

**FIGURE 7:**
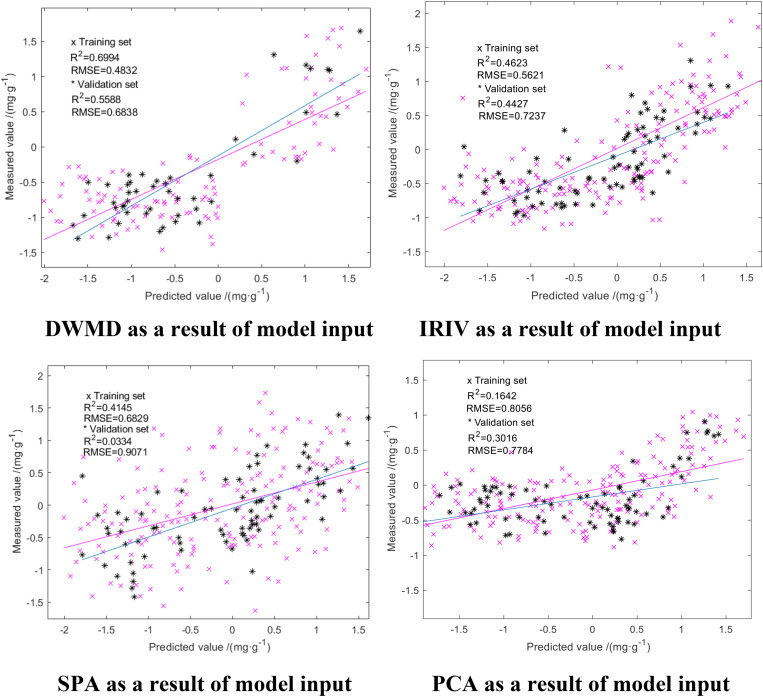
PLSR modeling results.

**FIGURE 8:**
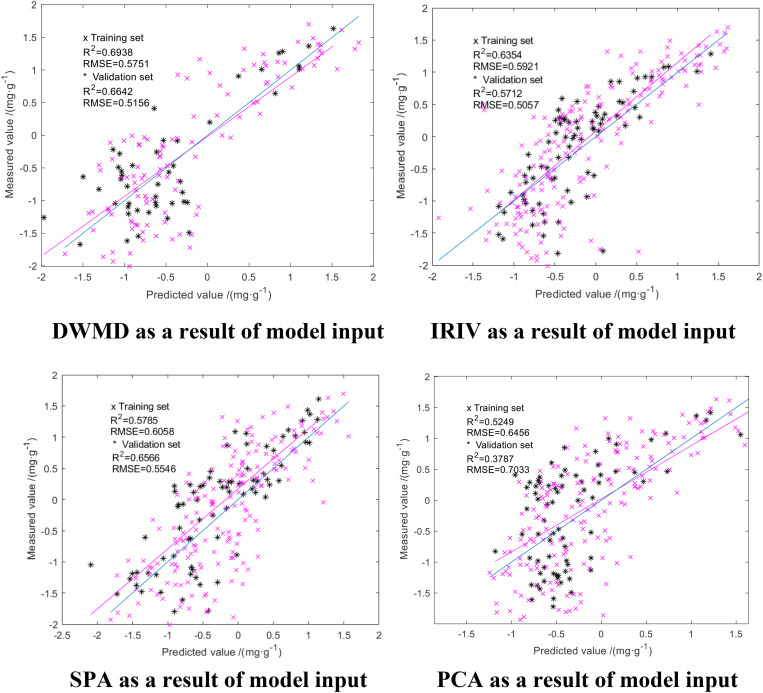
ELM modeling results.

We compared the GA-ELM with the PLSR and ELM models, which are all widely used in hyperspectral inversion models. The results are shown in Figures 7, 8. The same characteristic parameters as the GA-ELM model were selected as inputs, and the model parameters were adjusted to the optimal state.

From Figures 7, 8, it can be seen that among the PLSR prediction model test results of different dimension reduction methods, the PLSR prediction model built with the result of multiscale decomposition of the discrete wavelet had the highest accuracy; the R^2^ of the training set and the verification set was 0.6994 and 0.5588, respectively, and the RMSE was 0.4832 and 0.6838 mg/g, respectively. Among the ELM prediction model test results of different dimension reduction methods, the ELM prediction model built with the result of discrete wavelet multiscale decomposition had the highest accuracy. Overall, the R^2^ of the ELM training set and validation set were better than those obtained using the PLSR prediction model, but compared with the GA-ELM prediction model, the decision coefficient R^2^ of the GA-ELM prediction model was the highest and the RMSE was the lowest. By comparative analysis, the predictive ability and model stability of the GA-ELM prediction model were better than those of the inversion model established by the other two methods.

## Discussion

The use of hyperspectral technology for rice N nutrition monitoring is one of the important methods to guide the precise fertilization of rice and is also the focus of research in precision agriculture (Prasanna et al., 2012). We established a mathematical model of the quantitative relationship between hyperspectral reflectance and the N content of rice leaves so that when using spectral information as an input, an inversion model can be used to obtain the N content of rice in an area and thus provide a method to evaluate the nutritional status of the rice (Ata-Ul-Karim et al., 2017; Du et al., 2018). When using the inversion N content of rice leaves to guide field fertilization, the standard N content of rice in the same period is also needed as a reference for evaluating the N nutrition deficit in the rice (Tsujimoto et al., 2019). Choosing a suitable reference standard is of great significance for the rapid and accurate determination of the proper fertilization for rice fields.

The combination of the critical N concentration curve and target yields was used as a reference value for standard N content in some related studies (Feng et al., 2016). The advantage of this method is that it has a strong agronomic mechanism and only requires precise inversion of rice N concentration and rice dry matter mass (Din et al., 2017). The method used in this study was based on the construction of a demonstration field of the standard rice cultivation pattern; the rice N content in the same period in the demonstration field was used as the evaluation criterion (Maes and Steppe, 2019). Although the critical N concentration method has a strong agronomic mechanism, the critical N concentration curves for different rice varieties and different growing areas need to be constructed uniquely, which is difficult for researchers who lack basic agricultural background information (Aasen et al., 2014). In this study, the quantitative relationship between the hyperspectral profile and the N content of the rice was modeled by collecting the hyperspectral and N content of both standard and experimental fields and calculating the difference between the hyperspectral reflectance and the N content of the two. The calculated difference in the hyperspectral reflectance was the input in an inversion model used to obtain the scarcity value of N content.

The method for obtaining a standard hyperspectral reflectance and N content as used in this study is relatively easy in practice. However, compared with the reference standard values established by the critical N concentration method, there are certain shortcomings in the determination of standard hyperspectral reflectance and standard N content, and the selection of standards is more based on statistical methods. In future research, we must focus on how to combine the critical N concentration method with the standard rice production model to collect the reference hyperspectral reflectance and reference N content of rice at different reproductive stages in order to evaluate the N nutrient deficit of rice more simply, quickly, and accurately.

In this study, we established an inversion model of N content differential in rice leaves. In terms of data reduction, this study used discrete wavelet multiscale decomposition, continuous projection, principal component analysis, and IRIV methods to downscale the spectra. When the results of these four downscaling methods were used as modeling inputs for ELM and GA-ELM, the wavelet approximation coefficients obtained based on discrete wavelet multiscale decomposition had the highest modeling accuracy. The discrete wavelet multiscale decomposition discretizes the scales and displacements in the continuous wavelet transform and combines the distribution of the wavelet signal energy on each scale, thus compressing the spectral signal dimensions, reducing the number of characteristic bands, and highlighting the spectral profile information. The wavelet approximation coefficient is used to reconstruct the signal, and the reconstructed spectral signal retains the original spectral information to the maximum extent. In building the model, since the relationship between the wavelet approximation coefficient and leaf N deficiency is more suitable to be fitted by a non-linear exponential model, the high value of leaf N content was severely underestimated when using PLSR for the linear regression of leaf N content, thus reducing the overall prediction accuracy of the model and resulting in a large RMSE error. Therefore, when modeling by ELM and GA-ELM methods, the modeling accuracy of wavelet approximation coefficients based on discrete wavelet multiscale decomposition was the highest, while the modeling accuracy of wavelet approximation coefficients based on wavelet multiscale decomposition was the lowest when modeling by the PLSR methods. The modeling accuracy of ELM and GA-ELM was higher than that of PLSR.

The GA-ELM models were better than the PLSR and ELM models because the GA-ELM and ELM models train neural networks with non-linear function input and output data so that the trained network can predict the non-linear function output, which can effectively explain the non-linear problem. The inversion accuracy of the GA-ELM models was better than that of the ELM models because the optimization training of GA can assign the initial weights of the ELM and thus reduce the problem of randomly generating weights for the ELM, which improves the model accuracy, stability, and generalization.

This study proposed a hyperspectral detection method for the diagnosis of nitrogen deficiency in rice fields. Spot-application of fertilizer in the field is very important for rice production, and existing studies mainly focus on nitrogen content testing, which can only obtain the current nitrogen level in the rice and does not indicate whether there is a nitrogen deficiency in the rice. By introducing the concept of standard nitrogen content and standard hyperspectral reflectance, this study established the difference between standard nitrogen content and sample nitrogen content, and calculated the difference between the corresponding hyperspectral reflectance. We established a quantitative relationship model between the nitrogen difference and hyperspectral difference that can be used to calculate the nitrogen deficiency of rice by obtaining hyperspectral reflectance. This can be used as a basis for decision making for field fertilizer spot-application based on nitrogen deficiencies in rice. The standard spectra and standard nitrogen content established in this study were obtained based on a statistical approach that has some shortcomings. However, this method can be used as a basis for further research. We believe that this manuscript is of practical significance.

## Conclusion

In this study, hyperspectral reflectance was determined by disruption sampling of rice crop stage japonica leaves, and the N content was measured in the laboratory after “greening-drying-grinding.” A database of differential spectral reflectance and differential N content data was constructed for the determination of the standard spectral reflectance and standard N content based on the principle of the highest yield. The spectral reflectance difference was treated by four methods: SPA, DWMD, PCA, and IRIV. The downscaled results were modeled using the PLSR, ELM, and GA-ELM methods. The results showed that the GA-ELM prediction model using the wavelet approximation coefficients obtained from the DWMD was the most accurate, with the training and validation sets >0.68 and the RMSE < 0.6 mg/g. The present study was carried out to provide a new approach to assess N scarcity during the rice reproductive period.

## Data Availability Statement

The raw data supporting the conclusions of this article will be made available by the authors, without undue reservation.

## Author Contributions

FY and SF conceived and designed the experiments. WD, DW, and ZG performed the experiments. FY analyzed the data. FY and SF wrote the original manuscript. FY, ZJ, YC, and TX reviewed and edited the original manuscript. All authors contributed to the article and approved the submitted version.

## Conflict of Interest

The authors declare that the research was conducted in the absence of any commercial or financial relationships that could be construed as a potential conflict of interest.
